# CEAF: Capsule network enhanced feature fusion architecture for Chinese Named Entity Recognition

**DOI:** 10.1371/journal.pone.0332622

**Published:** 2025-10-07

**Authors:** Siyu Ma, Guangzhong Liu, Yangshuyi Xu

**Affiliations:** College of Information Engineering, Shanghai Maritime University, Shanghai, China; Dr. NGP Institute of Technology, INDIA

## Abstract

Chinese Named Entity Recognition (NER) is a fundamental task in the field of natural language processing, where achieving deep semantic mining of nested entities and accurate disambiguation of character-level boundary ambiguities stands as its core challenge. Existing methods, mostly based on the BiLSTM-CRF sequence labeling framework or Transformer attention mechanisms, have inherent limitations in modeling the hierarchical structural dependencies of nested entities and resolving semantic conflicts in overlapping character spans. To address challenges such as the lack of morphological markers, propagation of boundary ambiguities, and insufficient geometric modeling in the feature space, we propose a novel multi-stage neural architecture—the CEAF model, a specialized neural framework tailored for Chinese NER tasks. The architecture leverages BERT-derived subword embeddings to capture character-level contextual representation and incorporates BiLSTM to model position-sensitive sequential patterns. Meanwhile, to effectively tackle the complex challenges of boundary uncertainty and nested entity composition, the CEAF model innovatively introduces the Deep Context Feature Attention Module (DCAM). This module pioneeringly integrates capsule routing protocols with position-aware attention mechanisms, processing information through dual parallel paths: on one hand, it leverages the powerful spatial relationship modeling capability of capsule networks to clearly parse the hierarchical structure and part-whole relationships between entities; on the other hand, it utilizes position-aware attention to focus on key positional information, dynamically adjust the attention to different positional information, accurately locate entity boundaries, effectively resolve boundary ambiguity, and achieve efficient and accurate modeling of nested entity structures. In addition, the Adaptive Feature Fusion Network (AFFN) effectively bridges the semantic gap between global contextual coherence and local boundary precision by selecting more discriminative fusion features. Generalization experiments on three Chinese benchmark datasets and one English dataset demonstrate that the CEAF model outperforms baseline models. Visualization analysis further verifies the modeling capability of the CEAF model, providing new insights into geometric deep learning approaches for Chinese NER.

## 1 Introduction

In the era of social media, Chinese online texts are experiencing exponential growth, with embedded entity names and event feedback providing crucial evidence for effectiveness evaluation [38]. However, the hierarchical nested entity structures requiring dynamic routing protocols, non-canonical linguistic phenomena with adversarial noise patterns in user-generated content, and heterogeneous feature fusion challenges in multimodal data streams pose significant challenges to entity recognition [12,16–18]. As a basic task in natural language processing, NER can identify key entities such as names of people, organization names, and places from unstructured text. It has important application value in public opinion monitoring, knowledge graph construction, intelligent customer service and other fields, and provides important support for effect analysis and monitoring.

In the named entity recognition task, Chinese and English show significant differences due to differences in language characteristics [3,6–8]. From the perspective of boundary determination, Chinese NER relies on attention-based contextual semantic modeling and sliding window enumeration with dynamic programming, while English NER leverages explicit tokenization rules and case-sensitive orthographic features to achieve deterministic segmentation, resulting in lower computational complexity for boundary recognition [9,10]. The presence of nested entities further exacerbates this complexity. Nested entities refer to phenomena where one entity contains another entity of the same or different types within text boundaries. For nested entity processing, Chinese requires span-based graph neural networks or hierarchical boundary-aware modules to decode multi-layer nested structures, whereas English entities typically follow flat annotation schemas, with mainstream methods assuming entity independence by default [21,27]. This hierarchical structure violates the traditional sequence labeling assumption of “non-overlapping independent entities,” demanding models to not only recognize entity boundaries but also parse inclusion relationships. In domain adaptation, Chinese NER necessitates domain-specific dictionary injection to mitigate pre-trained model knowledge gaps, while English models benefit from parameter-efficient fine-tuning enabled by large-scale corpus priors during cross-domain transfer. The strong reliance of Chinese NER on domain-specific dictionaries results in notable drawbacks compared to English NER. Firstly, its generalization capability is weak. Chinese terms lack consistent standardization and vary significantly across domains; when switching to a new domain without a corresponding dictionary, recognition accuracy drops drastically. In contrast, English NER relies on standardized orthography and semantic reasoning, enabling more flexible cross-domain transfer. Secondly, dictionary maintenance incurs high costs, as new Chinese terms require continuous updates by domain experts, whereas English neologisms can be inferred through word-formation rules, reducing manual dependency. Additionally, it is prone to data sparsity issues, with low-frequency terms struggling to acquire semantic features. Over-reliance on dictionaries also leads to mechanical matching that ignores contextual semantics, making it more challenging for Chinese NER to achieve recognition accuracy than English NER.

**Fig 1:**
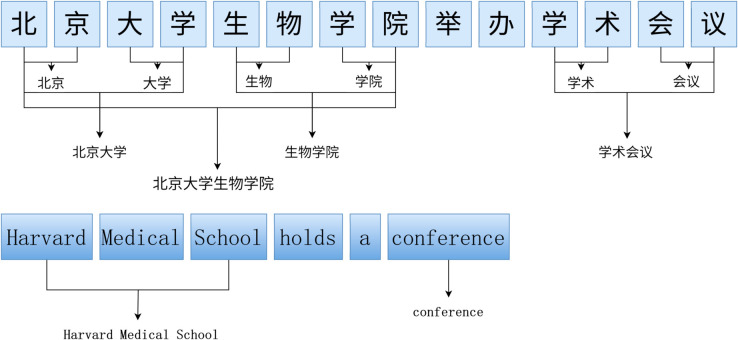
Comparison of NER Cases between Chinese and English.

Take the sentence in Fig 1 above as an example. These two sentences are very similar in meaning, but the difficulty of recognition differs significantly due to inherent linguistic divergences in entity composition rules. When recognizing Chinese named entities, the system must simultaneously resolve span overlaps in nested entity structures—for instance, identifying “Peking University” as an independent institution while recognizing “Peking University School of Life Sciences” as a nested institution entity containing compositional semantics. This structural dependency relies on hierarchical semantic parsing of lexical collocations between “university” and “school.” Additionally, the absence of word boundary delimiters in Chinese necessitates context-aware tokenization for character sequences like “Beijing,” “university,” “biology,” and “school,” requiring boundary disambiguation through bidirectional semantic constraints. In contrast, the English example “Harvard Medical School holds a conference” benefits from explicit morphological markers: Spaces naturally segment the institution name “Harvard Medical School” as a flat-structured entity without requiring nested resolution. Orthographic features like capitalization and whitespace substantially reduce boundary detection complexity.

In the field of Chinese named entity recognition, researchers have proposed various solutions addressing distinct technical challenges. 1) For handling blurred entity boundaries due to Chinese’s separator-free nature, a radical-enhanced BiLSTM-CRF architecture leverages morphological decomposition of Han characters through bidirectional LSTM networks, while CRF-based label transition constraints optimize boundary detection in structured texts [9,10]. 2) To address nested entity recognition, the SAT-CNER model implements a dynamic span enumeration strategy with sliding window operators, enhanced by adversarial training with gradient perturbation to improve structural robustness [11]. 3) To address the challenge of domain-specific dictionary injection, the dynamic dictionary fusion framework converts domain terms into learnable vector representations. It dynamically adjusts the weight allocation between dictionary features and contextual semantics through an attention mechanism, effectively avoiding context misjudgment caused by mechanical matching. 4) The BERT-BiLSTM-CRF framework combines contextualized embeddings from transformer layers with sequential pattern learning via gated recurrent units, forming a hierarchical representation learning framework constrained by CRF-based state transitions [13,14]. 5) For vertical domain adaptation, a pyramidal CNN architecture hierarchically fuses multi-scale BERT representations through shallow convolutional filters for local n-gram patterns and deep dilated convolutions for global context integration, complemented by structured pruning for parameter sparsification and 8-bit quantization to reduce deployment overhead [15].

However, existing methods still have multi-dimensional limitations. 1) Although the radical-based model performs robustly in regular texts, it lacks the ability to parse the semantics of phonetic abbreviations and new words on social media, and it is difficult to capture the language characteristics of non-standard expressions [19,20]. 2) Although the dynamic sliding window mechanism improves the recognition rate of nested entities, it is inefficient in capturing entities that jump across long distances across sentences, and the window enumeration causes the computational complexity to grow exponentially [22,23]. 3) Although the deep integration of pre-trained models and CRF strengthens semantic modeling, the huge number of parameters of BERT causes a surge in training energy consumption [26]. 4) Domain-specific dictionary injection still has notable limitations in Chinese Named Entity Recognition (NER). Dictionaries struggle to fully cover domain-specific terms, often failing to include emerging vocabulary, dialects, and other special expressions. Additionally, delayed updates mean new terms cannot be promptly incorporated. When processing cross-domain texts, integrating different dictionaries easily leads to term conflicts and misjudgments. Furthermore, over-reliance on dictionary matching ignores contextual semantics, resulting in inaccurate entity judgments in complex contexts and undermining recognition performance.5)Vertical domain models are limited by the scarcity of professional annotated data, and the entity recall rate is generally less than 40%, with significant performance degradation during domain migration due to the semantic gap [24,25]. 6) Although lightweight technology improves deployment efficiency, its dependence on large-scale annotated data is fundamentally inconsistent with the needs of low-resource scenarios, making it difficult to support application expansion in subdivided fields such as dialects and small languages [28]. The deeper problem is that Chinese NER has not yet established a unified annotation standard, with differences in entity type systems across domains limiting the model’s generalization ability, while cutting-edge directions such as multimodal knowledge fusion and unsupervised pre-training still need breakthrough progress.

In social media, user-generated content is massive and unstructured, containing critical information that urgently requires extraction—this underscores the importance of Chinese Named Entity Recognition (NER). It supports public opinion monitoring to capture public attitudes and enables the construction of social network graphs for analyzing social relationships. However, significant challenges persist: non-standard user expressions with numerous abbreviations and errors, the lack of distinct word boundaries in Chinese, and the continuous emergence of new vocabulary and buzzwords, all posing immense difficulties for recognition tasks.

To address these issues and limitations, particularly in processing nested entities and entities within informal texts, we innovatively propose the CEAF model. Its core innovation lies in constructing a dynamic collaborative semantic parsing framework for Chinese NER, breaking free from the constraints of traditional models that rely on simple module stacking or single-dimensional feature extraction. The model’s key breakthrough lies in resolving the critical contradictions in Chinese NER: through the design of a “structure-semantics” dual-dimensional dynamic collaboration mechanism, it achieves deep coupling between fine-grained internal entity features and global contextual semantics. Instead of treating entity structure modeling and contextual semantic capture as isolated processes, it establishes a mutual feedback relationship—enabling the accurate extraction of entity morphological features to dynamically respond to changes in contextual semantics, while contextual semantic capture reciprocally enhances the refined parsing of internal entity structures. More importantly, the model innovatively incorporates an adaptive semantic-aware fusion mechanism, eliminating the rigidity of traditional fixed-weight fusion. It dynamically adjusts feature aggregation strategies based on text semantic complexity, allowing precise alignment between local entity features and global contextual information in complex scenarios. This collaborative framework fundamentally enhances the model’s ability to tackle core challenges such as boundary ambiguity caused by Chinese’s lack of explicit separators, complex hierarchical relationships in nested entities, and variable semantic ambiguity scenarios, achieving an essential leap from “module concatenation” to “mechanism collaboration”. The main contributions of this paper are summarized as follows:

1) This paper innovatively proposes the CEAF model architecture, a dynamically collaborative feature parsing system designed to address nested and irregular entity recognition in unstructured social media texts. Breaking free from traditional module stacking limitations, it organically decouples semantics of BERT, the sequence pattern of Bi-LSTM, and the geometric representation of the capsule network.Via the AFFN module, it enables dynamic multi-dimensional feature aggregation, adapting to non-standard expressions and emerging vocabulary to significantly boost entity recognition accuracy.

2) We propose the DCAM contextual feature attention module, specifically designed to resolve semantic ambiguity in Chinese NER within informal texts. The module uses adaptive pooling layers in parallel to capture global statistical features, combines 1D convolution to extract local context associations, and implements feature adaptive calibration through gated units. The multi-granularity fusion architecture dynamically aggregates global and local features to generate position-sensitive context representations.

3) We propose a feature enhancement network (AFFN) based on an adaptive feature fusion mechanism. This module breaks through the limitations of traditional fixed-pattern fusion. By constructing a dynamic feature interaction mechanism, it deeply explores the potential semantic correlations among multi-source heterogeneous features and can adaptively adjust fusion strategies based on text semantic complexity. This design enables the intelligent fusion of cross-level features, effectively enhancing the model’s ability to recognize nested entities and entities with blurred boundaries, and providing key support for solving the core challenges in Chinese named entity recognition.

The structure of this paper is as follows: Sect 2 is Related work, which mainly reviews the research progress in the field of named entity recognition. Sect 3 introduces the model structure proposed in this paper in detail. Sect 4 is the experiment and result analysis to prove the effectiveness and interpretability of the model. Sect 5 is the conclusion part, which comprehensively reviews the research work of this paper.

## 2 Related works

### 2.1 Chinese Named Entity Recognition

The development of Chinese named entity recognition methods has gone through a process from traditional rule-driven to deep learning-led. Early named entity recognition mainly relied on rule templates and statistical models, such as rule-based methods based on dictionary matching and statistical methods like hidden Markov models (HMM) [29,31]. These methods perform well in structured texts, but they have poor generalization ability when facing informal texts on social networks and are sensitive to data noise. With technological breakthroughs, in 2014, Tianchuan Du et al. proposed a Chinese NER model based on deep learning, using convolutional neural networks (CNNs) to extract features, which marked the beginning of deep learning being applied to Chinese NER tasks. Ashish Vaswani et al. (2017) proposed the Transformer model [41], a model based on the self-attention mechanism, which was subsequently widely used in NER tasks. Chinese NER methods based on deep learning have become the mainstream. This method mainly uses neural network models to extract text features and predict entity boundaries and types [32,33]. Convolutional neural networks extract local features through convolutional layers and pooling layers, and are suitable for capturing local patterns in text, while recurrent neural networks, especially long short-term memory networks and gated recurrent units, can process sequence data and pass previous text information through hidden states, making them suitable for processing variable-length text. Despite significant advancements in Chinese NER driven by deep learning technologies, this task still faces pressing challenges today, and continuously improving its accuracy holds crucial practical significance—many key application scenarios heavily rely on the precision of entity recognition. For instance, in public opinion monitoring, failure to accurately identify core entities such as people, organizations, and events in texts will lead to deviations in public attitude analysis, affecting the timeliness and relevance of decision-making responses. In intelligent customer service and dialogue systems, omissions in entity recognition may cause misinterpretation of user intentions, directly reducing service quality and user experience. In the field of financial risk control, misjudgment of entities such as enterprise names and transaction subjects could even pose potential risk hazards. The inherent lack of explicit word boundary markers in Chinese makes accurate entity boundary segmentation inherently challenging. Particularly in informal scenarios like social media, users often employ mixed expressions involving homophones, abbreviations, English phrases, or emojis, further exacerbating text irregularity. Meanwhile, the hierarchical relationships of nested entities are complex and variable; a single text segment may contain multiple nested or overlapping entity types, making it difficult for traditional models to effectively capture such multi-dimensional semantic associations. Additionally, the continuous emergence of new domain terminology and internet buzzwords outpaces the iteration speed of models, resulting in widespread inadequacy in recognizing out-of-vocabulary words. Moreover, the scarcity of annotated data and domain differences make models prone to performance degradation during cross-scenario migration. The combination of these issues means that Chinese NER still struggles to meet the demands for high precision and strong robustness in practical applications, remaining a critical subject to be addressed in the field of natural language processing. Improvements in accuracy directly impact the reliability of downstream tasks and the realization of application value.

### 2.2 BERT module

The BERT (Bidirectional Encoder Representations from Transformers) model [40], proposed by Google in 2018, is a pre-trained language model that significantly improves the accuracy of Named Entity Recognition (NER) through pre-training and fine-tuning. It employs a bidirectional Transformer encoder to achieve deep semantic understanding of text. The core innovation of BERT lies in its large-scale unsupervised text learning of bidirectional contextual semantic representations. During pre-training, BERT utilizes two key tasks: 1. Masked Language Model (MLM): Randomly masking portions of input tokens and predicting them based on contextual clues, forcing the model to learn bidirectional contextual dependencies; 2. Next Sentence Prediction (NSP): Determining whether two sentences are consecutive text segments to enhance the model’s comprehension of inter-sentence logical relationships. In NER tasks, BERT’s application addresses traditional methods’ heavy reliance on annotated data while demonstrating remarkable improvements in recognizing complex contextual patterns and novel entities. The model’s architecture – featuring multi-layer Transformer encoders with token/segment/position embeddings–enables it to capture nuanced semantic relationships through self-attention mechanisms and positional encoding.

### 2.3 Capsule Network

Capsule Networks (CapsNets) [42], proposed by Geoffrey Hinton’s team in 2017, address the limitations of conventional convolutional neural networks (CNNs) in image processing through vectorized “capsules” that replace scalar-valued neurons. Each capsule’s output vector encodes both the existence probability of an entity and its spatial attributes, enabling hierarchical modeling of complex object structures. The dynamic routing mechanism, as the core algorithm, coordinates information flow between lower-level and higher-level capsules by iteratively optimizing coupling coefficients through semantic compatibility evaluation [36]. In entity recognition tasks, lower-level capsules capture local lexical features whose vector outputs indicate potential entity membership while encoding semantic roles through directional components. Higher-level capsules integrate these features via dynamic routing, analyzing inter-word logical consistency to determine precise entity boundaries and types. This mechanism calculates semantic matching degrees between capsule layers through iterative weight adjustments, prioritizing coherent feature combinations while suppressing noise and ambiguous patterns. The squash function nonlinearly compresses vector magnitudes while preserving directional semantics, effectively modeling complex linguistic phenomena. CapsNets demonstrate particular efficacy in low-resource/noisy-data scenarios, such as identifying informally expressed entities in social media texts or adapting to new domains with minimal annotated samples. Despite computational complexity from high-dimensional transformation matrices, they offer an innovative NLP paradigm combining hierarchical semantic modeling with dynamic pathway optimization, balancing local feature extraction and global structural reasoning [37].

### 2.4 Attention mechanism

The attention mechanism is a computational paradigm inspired by biological selective attention processes [43], designed to enhance neural networks’ focus on task-critical features through dynamic weight allocation. Originating from bionics research on human visual perception systems, this mechanism was first formalized for neural machine translation (NMT) by Bahdanau et al., effectively addressing the long-range dependency limitations and encoder capacity constraints inherent in conventional recurrent neural networks (RNNs). Its operational principle involves computing inter-feature relevance scores to guide computational resources toward discriminative regions within input data [34,35].

The mechanism’s technical innovation manifests in two key aspects: dynamic adaptability and architectural transparency. Unlike static parameter matrices in traditional convolutional/fully-connected layers, attention weights are dynamically adapted based on input context, enabling flexible modeling of intricate semantic relationships. In Transformer architectures, sequence-level attention captures extended contextual dependencies through all-pair positional relevance computations, while multi-head attention leverages parallel projection subspaces to extract complementary feature representations. This dual mechanism achieves simultaneous preservation of sequential relationships and parallel computation efficiency, fundamentally overcoming RNNs’ sequential processing constraints.

### 2.5 Feature fusion

As a pivotal technique for enhancing model representational capacity, feature fusion aims to construct more discriminative joint feature representations by integrating multi-source features across hierarchical levels, modalities, or scales. Its core objective addresses the informational constraints inherent in single-feature-space representations. Taking cross-lingual entity linking as a case study, this approach combines word-level cross-lingual embedding vectors with syntactic dependency tree structural features, while employing attention mechanisms to dynamically adjust interlingual alignment feature weights. Such implementation demonstrates a 19% improvement in entity disambiguation accuracy under low-resource language scenarios compared to baseline methods. Traditional methodologies primarily focus on early fusion and late fusion. Feature fusion enhancement, as a core deep learning optimization technology, fundamentally overcomes single-feature-space representation bottlenecks through multi-dimensional, multi-level feature interactions. It constructs joint feature spaces with enhanced semantic expressiveness via geometric constraints, dynamic weight adaptation, and cross-modal association strategies. This technical paradigm achieves progressive enhancement from low-level local features to high-level semantic abstractions, thereby improving model robustness and generalization in complex scenarios. A notable implementation is the dynamic Feature Pyramid Network (FPN), which optimizes scale adaptability in object detection through top-down fusion of high-level semantic features with low-level spatial details.

## 3 Model

### 3.1 Overall model architecture

**Fig 2:**
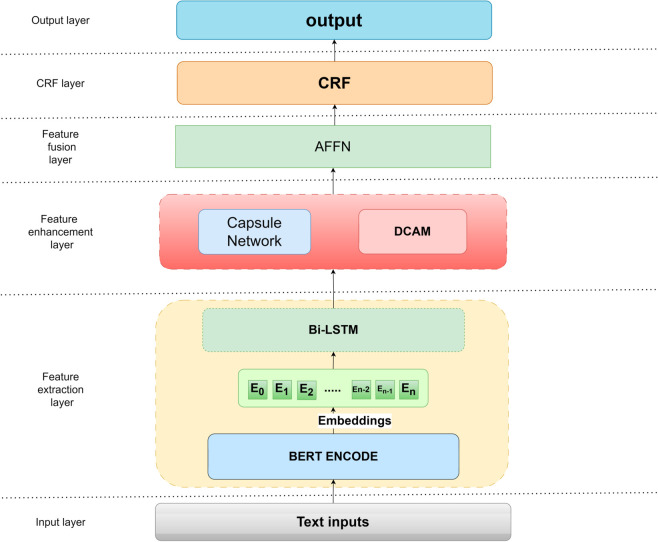
The overall framework of the CEAF model proposed in this paper.

The CEAF (Capsule Network Enhanced Feature Fusion Architecture) model we proposed is shown in Fig 2.

The CEAF model designs a multi-level collaborative architecture to address the core difficulties of Chinese NER, and improves entity boundary judgment and semantic parsing capabilities through hierarchical feature extraction and dynamic fusion mechanisms. The model first uses the BERT pre-trained language model to generate character-level dynamic semantic representations, and uses the Transformer’s bidirectional attention mechanism to capture long-distance contextual dependencies, effectively solving the ambiguity problem of Chinese polysemy. Subsequently, the sequence is modeled using the Bi-LSTM network, and its gating mechanism can accurately capture boundary features such as entity prefixes and suffixes. The feature enhancement layer innovatively constructs a parallel path between the capsule network and the dynamic contextual attention DCAM module: the capsule network establishes a hierarchical association between character components and the overall entity through an iterative routing algorithm to enhance the recognition ability of nested entities; the DCAM recalibrates the contextual features based on position-sensitive attention weights to highlight the semantic contribution of the core components of the entity. The final adaptive feature fusion network (AFFN) dynamically integrates the global semantics of BERT, the sequence regularity of BiLSTM, and the structural features of the enhancement layer through a gating mechanism to achieve optimal weighting of multi-granular information. In the decoding stage, the CRF layer is used to constrain the label transfer path, and the Viterbi algorithm is used to decode the entity sequence that conforms to the grammatical rules, which significantly improves the rationality of the transfer probability between labels.

This section will explain our model from three aspects: Sect 3.2 Feature extraction, Sect 3.3 Feature enhancement and feature fusion, and Sect 3.4 Entity recognition.

### 3.2 Feature extraction and representation learning

#### 3.2.1 BERT layer.

In this paper, the BERT layer, as the underlying feature encoder, undertakes the core task of extracting deep semantic representations from the original text. Its workflow can be summarized as three stages: embedding mapping, context encoding, and feature transfer. Through the multi-level abstraction capabilities of the pre-trained language model, it provides dynamic feature expressions rich in grammatical structure and semantic relations for the upper-layer Bi-LSTM, feature enhancement module, and CRF. The core innovation of BERT lies in its bidirectional encoding mechanism. This bidirectional training method enables BERT to capture the complete semantic information of words in a sentence, thereby significantly improving the model’s ability to understand complex contexts. By stacking multiple layers of Transformer encoders, BERT extracts deep semantic features of the text layer by layer, where the self-attention mechanism allows the model to dynamically capture the strength of association between words in different positions, as shown in Eq (1).

$$Attention=softmax\left(\frac{QK^{𝖳}}{\sqrt{d_{h}}}\right)V$$ (1)

**Fig 3:**
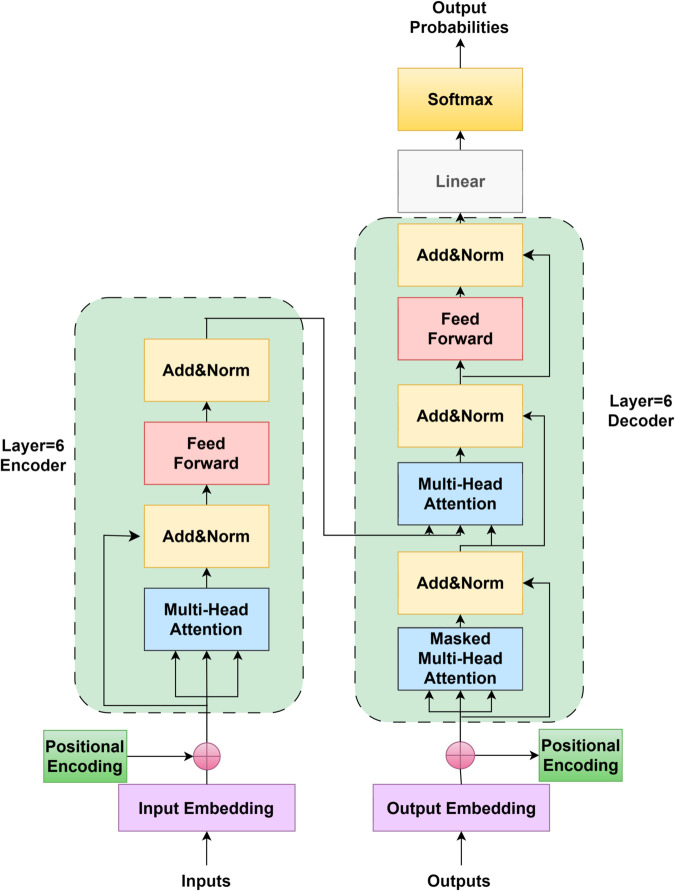
Transformer model architecture.

The Transformer model can achieve deep and fine-grained extraction of feature representations. Its model structure is shown in Fig 3. Each layer contains two core modules: self-attention and feed-forward neural network (FFN). The self-attention mechanism can dynamically capture the association strength of tokens at different positions in the text, enabling the modeling of long-distance semantic dependencies; the FFN deepens the expression of local features through non-linear transformations. With residual connections alleviating the gradient vanishing problem and layer normalization stabilizing the input distribution, features continuously deepen semantic abstraction during layer-by-layer transmission—evolving from basic morphological features to high-level semantic features. Finally, they are output as context vectors of each subword and passed to the Bi-LSTM layer, laying a solid foundation for sequence modeling.

At the base layer, the pre-trained BERT embeddings dynamically encode character-level semantics and global contextual dependencies. This layer solves the polysemy problem by generating context-sensitive representations for each Chinese character, using a bidirectional Transformer mechanism to capture long-range dependencies. The output is a sequence of context vectors $H_{BERT}=\{h_{1},h_{2},\cdots ,h_{n}\}$, where $h_{i}\in {\mathbb{R}}^{d_{BERT}}$ corresponds to the *i*-th character in the input text. Convert tokens to input_ids through the vocabulary, and map input_ids to a vector ${𝔼}_{token}\in {\mathbb{R}}^{n\times d}$, where d=768 is the hidden layer dimension. ${𝔼}_{pos}\in {\mathbb{R}}^{n\times d}$ represents the position information of the encoded token. ${𝔼}_{seg}\in {\mathbb{R}}^{n\times d}$ is used to distinguish sentences A and B. Add the three together to get $E\in {\mathbb{R}}^{n\times d}$ as the final input embedding matrix, as shown in Eq (2).

$$𝐄={𝐄}_{token}+{𝐄}_{pos}+{𝐄}_{seg}$$ (2)

#### 3.2.2 Bi-LSTM layer.

**Fig 4:**
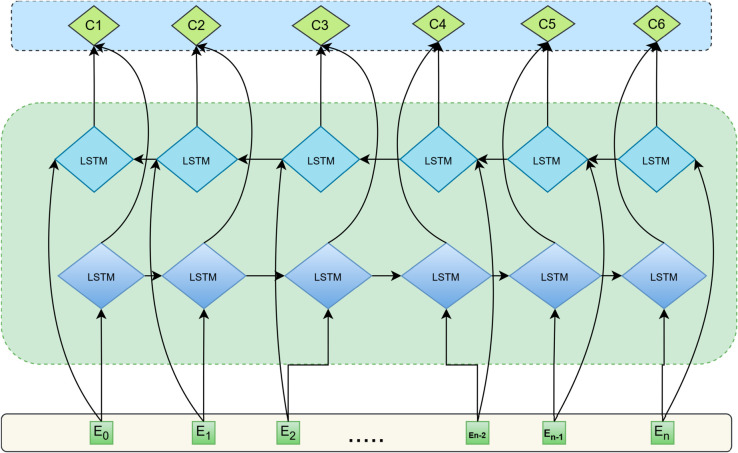
Bi-LSTM architecture.

Bi-LSTM(Bidirectional Long Short-Term Memory) is a deep learning model widely used in sequence labeling tasks. Its core idea is to combine two independent LSTM layers to process the input sequence from the forward and reverse directions. The forward LSTM layer processes the sequence in chronological order to capture the dependency between the current moment and the previous moment; while the reverse LSTM layer processes the sequence in reverse chronological order to capture the dependency between the current moment and the next moment. At each time step, the outputs of the forward and reverse LSTM layers are fused by vector concatenation to generate a feature representation containing complete context information. This bidirectional structure is shown in Fig 4 below.

In this paper, Bi-LSTM is used as the middle layer of the model, receiving the context-related feature representation output by the BERT layer, and is responsible for further modeling the sequential dependencies of the text and enhancing the model’s ability to understand long-distance contexts. Leveraging its unique bidirectional sequential modeling capability, Bi-LSTM not only can more precisely capture and model the hidden sequential dependencies in the text but also effectively enhances the model’s ability to understand semantic associations across long-distance contexts. This provides the subsequent feature enhancement layer and decoding layer with feature support that is more logically sequential and contextually complete, thereby further improving the overall model’s accuracy in recognizing entity boundaries and semantic relationships.

**Fig 5:**
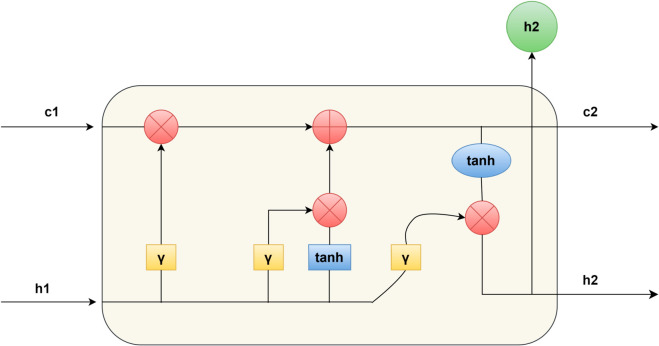
The network structure of the LSTM.

The internal structure of LSTM is shown in Fig 5. It includes four gating mechanisms: forget gate, input gate, update gate, and output gate. The forget gate controls the information to be forgotten in the cell state, the input gate determines the storage of new information, the update gate updates the long-term memory unit, and the output gate controls the output of the current hidden state. As shown in formulas Eqs (3)–(8):

$$f_{t}=\gamma (W_{f}\cdot [h_{t-1},x_{t}]+b_{f})$$ (3)

$$i_{t}=\gamma (W_{i}\cdot [h_{t-1},x_{t}]+b_{i})$$ (4)

$${\tilde{C}}_{t}=\tanh(W_{C}\cdot [h_{t-1},x_{t}]+b_{C})$$ (5)

$$C_{t}=f_{t}⊙C_{t-1}+i_{t}⊙\tilde{C_{t}}$$ (6)

$$o_{t}=\gamma (W_{0}\cdot [h_{t-1},x_{t}]+b_{o})$$ (7)

$$h_{t}=o_{t}⊙\tanh(C_{t})$$ (8)

Where r is the sigmoid function, $W_{f}\in {\mathbb{R}}^{h\times (h+d)}$, $b_{f}\in {\mathbb{R}}^{h}$, ⊙ represents element-by-element multiplication, and W*, b* represent the weight matrix and bias term.

In the feature extraction task, Bi-LSTM can use context information to identify nested entities and new entities. The combination of Bi-LSTM and BERT can effectively extract features and pass them to the next layer of structure.

### 3.3 Feature enhancement and feature fusion

In Chinese NER, the recognition of nested entities requires the model to capture multiple levels of semantic information at the same time. The noise and informality in Chinese text also increase the difficulty of entity recognition. Traditional models often have difficulty in effectively processing such complex structures. In order to address this limitation, we introduced two parallel subcomponents in the feature enhancement module: capsule network and DCAM module, and used AFFN module to adaptively fuse the enhanced features. When the Bi-LSTM layer transfers the extracted context features to this layer, the two modules use different technologies to enhance the features of effective information. Finally, the features extracted by the two modules are passed to the AFFN module for adaptive fusion to enhance the recognition of nested entities and irregular entities. We will explain it in detail below.

#### 3.3.1 Capsule network.

The core idea of capsule network is to represent different attributes of objects through capsules and model the relationship between features through dynamic routing mechanism. The basic unit of capsule network is capsule. The output of each capsule is a vector, whose direction represents the feature attribute and length represents the probability of the feature existence. Different from the traditional scalar output, the vectorized representation of capsule can describe the features more richly and enhance the expressive power of the model.

In the CEAF model, the capsule network receives the context-related feature representation output by the Bi-LSTM layer and captures the spatial relationship between features through a dynamic routing mechanism. The dynamic routing mechanism determines the connection weights between low-level capsules and high-level capsules through iterative calculations, thereby modeling the spatial relationship of features.

**Fig 6:**
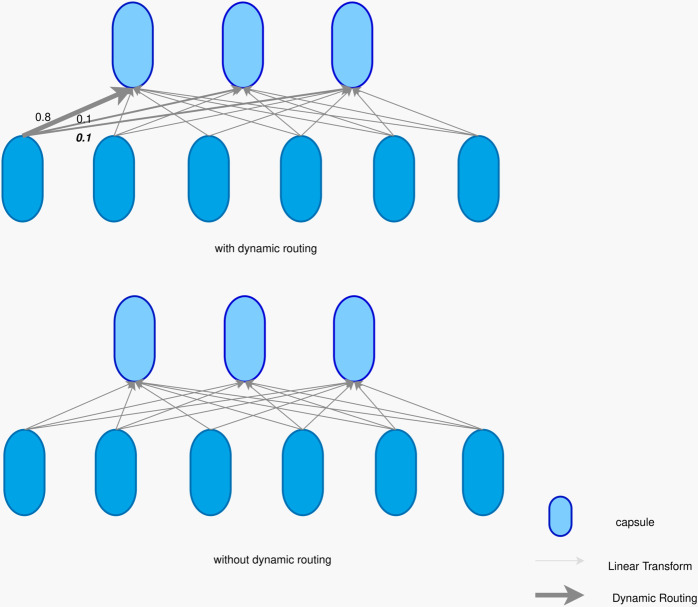
Dynamic routing mechanism.

The dynamic routing mechanism is the core innovation that distinguishes the capsule network from the traditional neural network. Its existence or not directly determines the model’s spatial relationship modeling capability, feature transfer efficiency, and task generalization performance. In a capsule network with dynamic routing, the low-level capsule iteratively adjusts the coupling coefficient to transfer the input vector to the high-level capsule that is “consistent” with its spatial attributes. This routing process simulates the bottom-up and top-down information integration mechanism in the biological neural system. If the dynamic routing is removed, the capsule network will degenerate into a static connection structure. At this time, the output of the low-level capsule is directly transferred to the high-level capsule with preset weights, similar to the fully connected layer of the traditional neural network. This static routing cannot dynamically adjust the information flow according to the input features, resulting in the model losing its equivariance to spatial transformations. The dynamic routing mechanism is shown in Fig 6 below.

Dynamic routing maps low-level capsules to high-level capsules by iteratively updating the coupling coefficient *c*_*ij*_. 1) Initialize the coupling coefficient *b*_*i*_ = 0, $c_{ij}=softmax(b_{ij})$. 2) Generate the prediction vector as shown in Eq (9). 3) The high-level capsule input *s*_*j*_ obtained by weighted summation of the prediction vector, and the modulus length is limited by the compression function. As shown in Eqs (10) and (11). 4) Update the similarity *b*_*ij*_ between the high-level capsule output and the prediction vector, as shown in Eq (12).

$${\;\;}^{1}{\hat{u}}_{j|i}=W_{ij}p_{i}$$ (9)

$$s_{j}=\sum_{i}c_{ij}{\hat{u}}_{j|i}$$ (10)

$$v_{j}=squash(s_{j})=\frac{∥s_{j}{∥}^{2}}{1+∥s_{j}{∥}^{2}}\cdot \frac{s_{j}}{∥s_{j}∥}$$ (11)

$$b_{ij}\leftarrow b_{ij}+v_{j}\cdot {\hat{u}}_{j|i}$$ (12)

Where $W_{i,j}\in {\mathbb{R}}^{n\times n}$ encodes the spatial relationship, the modulus of ${𝒱}_{j}$ represents the probability of entity existence, and the direction encodes the attribute.

The capsule network achieves feature enhancement by implementing multi-level semantic fusion through a dynamic routing mechanism. Character capsules encode basic semantics through bidirectional LSTM, vocabulary capsules integrate external dictionary information, and syntactic capsules capture long-distance constraints in dependencies. Dynamic routing suppresses the contribution of noise features through Leaky-SoftMax, and prioritizes local features with consistent spatial relationships to aggregate into high-level entity capsules.

#### 3.3.2 DCAM module.

**Fig 7:**
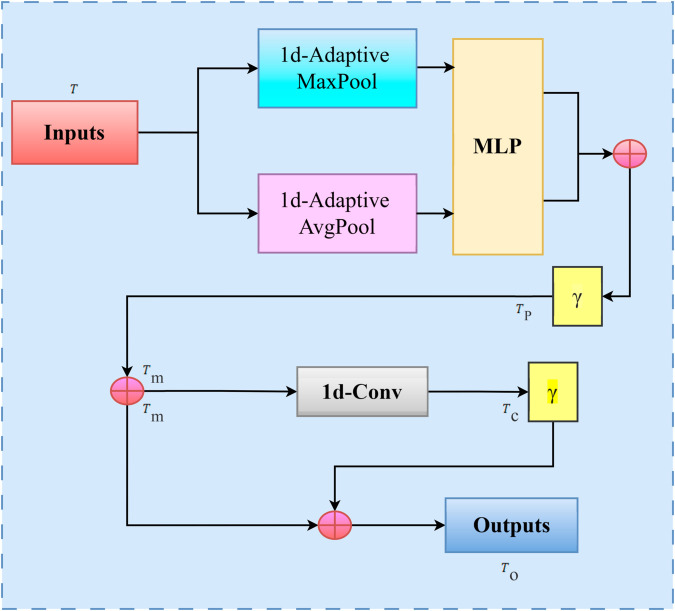
The structure of DCAM module.

The module structure of the DCAM (Deep context feature attention module) is shown in Fig 7 below.

In the DCAM module, the key features T in the contextual features captured by the Bi - LSTM network is first learned and screened through one-dimensional adaptive maximum pooling and one-dimensional adaptive average pooling to obtain adaptive pooling attention features *T*_*p*_. The process is shown in Eq (13):

$$T_{p}=\gamma (MLP(AvgPool_{1}D(T))+MLP(MaxPool_{1}D(T)))\;=\gamma \left(W_{1}\left(W_{0}(T_{a\nu g}^{p})\right)+W_{1}\left(W_{0}(T_{max}^{p})\right)\right)$$ (13)

Where γ(·) is the sigmoid nonlinear activation function; MLP represents a multi-layer perceptron; *W*_0_ and *W*_1_ are weight matrices used to perform linear transformation on the input in MLP.

Multiplying the text input feature T and the adaptive pooling attention feature *T*_*p*_ gives the weighted pooling attention feature *T*_*m*_, as shown in Eq (14).

$$T_{m}=T*T_{p}$$ (14)

By performing a one-dimensional convolution operation on the weighted pooled attention feature *T*_*m*_, the keyword semantic features in the context key features can be fully captured to obtain the convolutional attention feature *T*_*c*_, as shown in Eq (15).

$$T_{c}=\gamma \left(f^{1\times 3}([AvgPool_{1}D(T_{m});MaxPool_{1}D(T_{m})])\right)\;=\gamma \left(f^{1\times 3}([T_{a\nu g}^{c};T_{max}^{c}])\right)$$ (15)

Among them, γ(·) represents the sigmoid nonlinear activation function; $f^{1\times 3}$ represents the one-dimensional convolution operation using a convolution kernel of size 1×3.

After obtaining the convolutional attention feature *T*_*c*_, it is multiplied by the weighted pooling attention feature *T*_*m*_, and the output feature representation *T*_*o*_ of the DCAM module is obtained, as shown in Eq (16).

$$T_{o}=T_{m}*T_{c}$$ (16)

Compared with domain-specific dictionary injection methods, the DCAM module demonstrates significant advantages in adaptability and flexibility. Traditional domain-specific dictionary injection often relies on manually pre-constructed domain vocabulary lists, strengthening known domain entity features through static matching or weight assignment. However, this approach is limited by the coverage and update speed of the dictionary. When facing newly emerging words, variant expressions in the domain, or cross-domain mixed texts, it is highly prone to missing feature capture due to unrecorded vocabulary. In contrast, through adaptive pooling attention mechanisms and dynamic convolutional feature enhancement, the DCAM module can automatically learn the weight distribution of key features entirely based on the contextual semantics of the input text, without relying on predefined domain vocabulary sets. It can not only accurately focus on core semantic components in the text that are highly relevant to the entity recognition task but also flexibly adapt to the language styles and expression habits of texts in different domains. This effectively avoids the problem of insufficient generalization caused by the static nature of vocabulary in dictionary injection methods, ultimately achieving dynamic and fine-grained capture of key features and contextual association information in complex Chinese texts, and providing more targeted feature support for subsequent entity recognition tasks. By using the DCAM module, the Chinese text context features extracted by the Bi - LSTM network layer can be effectively extracted at a deeper and finer level, thereby effectively capturing the key features and key context feature information.

#### 3.3.3 AFFN module.

**Fig 8:**
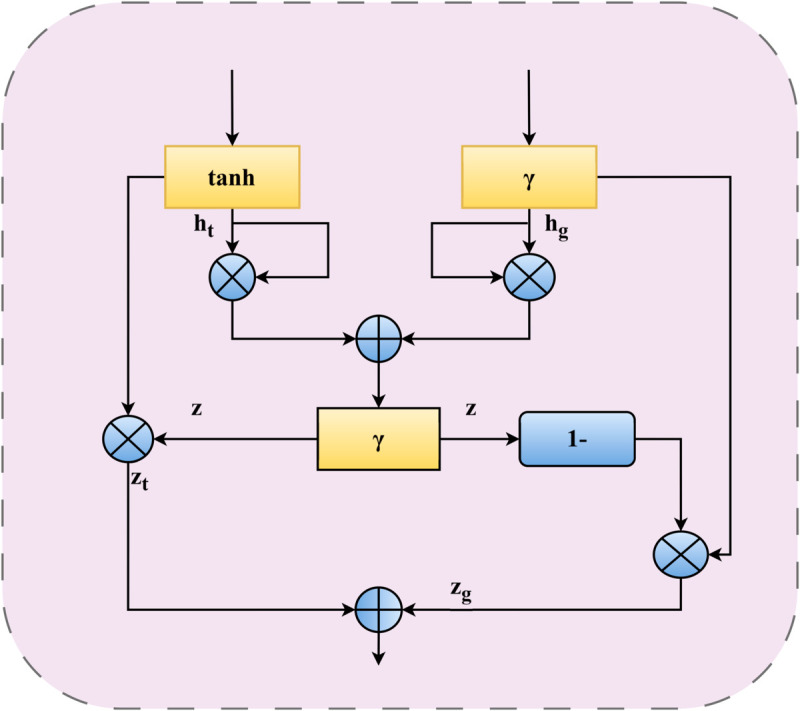
The structure of AFFN module.

The module structure of the AFFN (Adaptive Feature Fusion Network) is shown in Fig 8 below.

After obtaining the local spatial feature representation C encoded by the dynamic routing of the capsule network and the global dependency feature representation D extracted by the DCAM module, there may still be some redundant noise and cross-modal semantic biases in this feature information. We need to perform multi-granularity gating screening on it through the AFFN module to obtain more discriminative cross-level fused features for the model to make the final prediction. Inspired by the research of Y Xu et al. [1], we propose an innovative adaptive multi-modal feature fusion module, AFFN. This module can significantly enhance the semantic expression ability of the feature subspace and achieve in-depth adaptive fusion of features from different modalities.

The AFFN module conducts adaptive feature fusion calculations on two types of modal features: the text local spatial feature representation C and the global dependency feature representation D extracted by DCAM with feature attention. As a result, the fused feature representation f is obtained. The calculation process is as follows:

$$h_{c}=tanh(C)$$ (17)

$$h_{d}=sigmoid(𝔻)$$ (18)

$$h=(h_{c}*h_{c})+(h_{d}*h_{d})$$ (19)

z=sigmoid(h) (20)

$$z_{c}=z*h_{c}$$ (21)

$$z_{d}=(1-z)*h_{d}$$ (22)

$$f=z_{c}+z_{d}$$ (23)

In the above calculation process, first, according to Eqs (17), (18), and (20), in - depth and detailed information filtering is performed on the text feature C, the global dependency feature D extracted by DCAM, and their weighted - sum feature h respectively. Subsequently, by using Formulas Eqs (21)–(23), the input volume of feature information can be dynamically adjusted, thereby achieving the efficient integration of text features and self- attention features. As an innovative adaptive multi-modal feature fusion network, the AFFN module plays a crucial role in integrating and optimizing key features within the model architecture. It targets the local spatial feature representations encoded by the dynamic routing of the capsule network and the global dependency feature representations extracted by the DCAM module. Through a multi-granularity gating screening mechanism, it first performs in-depth and detailed information filtering on these two types of features as well as their weighted-sum features, effectively eliminating redundant noise and cross-modal semantic biases. Subsequently, by dynamically adjusting the input volume of feature information, it achieves efficient fusion of text features and self-attention features. For the highly challenging tasks of recognizing nested entities and informal entities in Chinese named entity recognition, the deep fusion capability of the AFFN module is particularly critical: it can accurately capture the entity boundary details in local spatial features and the contextual semantic associations in global dependency features, thereby effectively distinguishing the hierarchical relationships between nested entities and avoiding boundary misjudgments caused by feature confusion. Meanwhile, for issues such as semantic ambiguity and irregular formatting in informal expressions, its dynamic screening and fusion mechanism can strengthen the weights of core semantic features, weaken the interference of irrelevant noise, and enhance the model’s stability in semantic understanding and recognition of such non-standard entities, providing more precise cross-level feature support for the final entity recognition. This design innovatively breaks through the limitations of simple concatenation or weighting in traditional feature fusion. Relying on a deep adaptive fusion mechanism, it significantly enhances the semantic expression ability of the feature subspace, making the fused feature representations more discriminative, as they not only retain the detailed information of local spatial features but also integrate the contextual associations of global dependency features.

### 3.4 Entity recognition

CRF (Conditional Random Field), a probabilistic graphical model widely applied in sequence labeling tasks, can effectively model the global dependency relationships of label sequences. The core idea of CRF is to model the conditional probability distribution between the input sequence and the label sequence by defining feature functions and a transition matrix. In this experiment, CRF models the global dependencies of label sequences and handles nested entities and long entities, ensuring the consistency of the label sequence through global optimization. The conditional probability of the CRF model is calculated via the weighted sum of all feature functions. Given an input sequence x and a label sequence y of length n, the CRF model defines a conditional probability P(Y∣X), which is obtained by normalizing the sum of exponential functions over all possible label sequences, as shown in Eq (24):

$$P(Y|X)=\frac{1}{Z(X)}\exp(\sum_{t=1}^{T}\sum_{k}\lambda_{k}f_{k}(y_{t-1},y_{t},X,t))$$ (24)

where Z(X) serves as the partition function (normalization factor), ensuring the probability distribution sums to unity, as specified in Eq (25).

$$Z(X)=\sum_{Y^{\prime }}\exp\left(\sum_{t=1}^{T}\sum_{k}\lambda_{k}f_{k}(y_{t-1}^{\prime },y_{t}^{\prime },X,t)\right)$$ (25)

Feature function $f_{k}(y_{t-1},y_{t},X,t)$ characterizes the relationship between input sequence X, timestep t, and label transitions between *y*_*t*−1_ and *y*_*t*_.$\lambda_{k}$ is the weight of the feature function *f*_*k*_, which is learned from the training data. T is the length of the sequence.

In the model we proposed, the CRF is the last layer responsible for global optimization of the label sequence to ensure the rationality and consistency of entity recognition. The CRF receives the multi-level feature representation output by the capsule network and solves the optimal label sequence through the Viterbi algorithm. The Viterbi algorithm uses dynamic programming to find the label sequence that maximizes the global probability, thereby ensuring the rationality of entity recognition.

## 4 Experiments and analysis

To demonstrate the effectiveness of the CEAF model, this section is divided into four sections. In Sect 4.1, we introduce the evaluation metrics and hyperparameter settings used in this experiment; in Sect 4.2, we introduce the dataset used in this experiment; in Sect 4.3, a series of ablation studies are conducted on the CEAF model: in Sect 4.3.1, the number of capsules in the capsule network is ablated; in Sect 4.3.2, the convolution kernel size in the DCAM module is ablated; in Sect 4.3.3, the epoch is ablated; in Sect 4.3.4, the model variants of CEAF are ablated; in Sect 4.4, we conduct cross-language generalization experiments; in Sect 4.5, the CEAF model is compared with the SOTA model; All experiments in this paper are completed using RTX 4060 Ti graphics card and deep learning framework Pytorch.

### 4.1 Evaluation metrics and hyperparameter settings

In this experiment, we adopt accuracy, precision, recall, and F1 score as evaluation metrics to comprehensively assess the model’s performance in the Chinese NER task. Precision represents the proportion of true positive samples among all samples predicted as positive by the model; recall represents the proportion of true positive samples correctly predicted by the model among all actual positive samples; the F1 score is the harmonic mean of precision and recall, which can comprehensively reflect the model’s performance; accuracy evaluates the model’s overall performance across all samples. These metrics comprehensively reflect the model’s ability to identify entity boundaries and types, as well as handle non-standard texts. The formulas are shown in Eqs (26)–(29).

$$Accuracy=\frac{L\_correct}{L\_true}\times 100\%$$ (26)

$$Precision=\frac{E_{-}correct}{E_{-}all}\times 100\%$$ (27)

$$Recall=\frac{E\_correct}{E\_true}\times 100\%$$ (28)

$$F1=\frac{2\times Precision\times Recall}{Precision+Recall}\times 100\%$$ (29)

Where E_correct is the number of correctly predicted entities, E_true is the total number of predicted entities, E_all is the total number of entities, L_correct is the number of correctly predicted labels, and L_true is the total number of predicted labels.

Following the recommendations of Reference [1,38,39], we set the hyperparameters as shown in Table 1. The maximum sequence length is set to 128 to adapt to the characteristics of social media texts, the learning rate is set to 2e-5 and a linear decay strategy is adopted, and the dropout rate is kept at 0.1 to prevent overfitting. The training process uses mixed precision acceleration technology, the batch size is set to 16, the optimizer is Adam. All experiments were completed on a workstation equipped with an NVIDIA 4060Ti graphics card and implemented based on the PyTorch framework. To ensure the reliability of the results, each experimental configuration was repeated 3 times to take the average indicator.

**Table 1:** Hyperparameters.

Parameter name	Parameter value
Optimizer	Adam
Batch Size	16
Max_seq_length	128
Learning_rate	2e-5
Drop_out	0.1

### 4.2 Datasets

To comprehensively evaluate the performance of the CEAF model, this study selected three representative Chinese named entity recognition datasets and one typical English dataset: Toutiao, Weibo, MSRA, and Onto Notes 5.0. The results of entity recognition tasks were compared on the three Chinese NER datasets to verify the performance advantage of the CEAF model in the Chinese NER task. At the same time, a generalization experiment of the DCBA model was conducted on the English dataset to further prove the effectiveness and interpretability of the model.

1) Toutiao dataset: one of the widely used benchmark datasets in the field of natural language processing. Its core value lies in providing large-scale, multi-category Chinese short text samples, which are suitable for tasks such as text classification, entity recognition, and keyword extraction. This dataset is collected from news information on the Toutiao client and stored in a structured format. Each piece of data contains news ID, classification code, classification name, news title, and keywords.

2) Weibo dataset: This dataset contains user-generated content from the Sina Weibo platform, annotated with entity types such as names of people, places, and institutions. Its text has typical social media characteristics, including Internet slang, colloquial expressions, and non-standard grammatical structures, which can effectively test the model’s ability to process non-standard text.

3) MSRA dataset: as a benchmark dataset in the field of Chinese named entity recognition, it contains standard text in the news field. The introduction of this dataset helps to evaluate the generalization performance of the model on standard text;

4) OntoNotes 5.0 dataset: a multi-domain and multi-task corpus jointly constructed by the University of Pennsylvania, the Information Science Institute of the University of Southern California, and other institutions. Its core value lies in providing a unified semantic annotation framework across text types. The dataset defines 18 categories of fine-grained entities, including time, cardinality, events, legal terms, etc. The diversity of the four datasets covers texts from different fields and styles, which can fully verify the robustness and adaptability of CEAF under Chinese texts and its transferability under English texts. The datasets are shown in Table 2:

**Table 2:** Dataset.

Dataset	Training	Validation	Test
Toutiao	5375	2870	2733
Weibo	5200	510	480
MSRA	6750	1350	1193
OntoNotes 5.0	9987	1421	1377

### 4.3 Ablation experiment

#### 4.3.1 Impact of the number of capsules in capsule networks on model performance.

In this experiment, we use the Toutiao dataset as the research object, and observe the model’s ability to express Chinese nested entities and multi-type entities by dynamically adjusting the number of high-level capsules. If the number of capsules is insufficient, the model may be forced to compress different semantic entities into the same capsule, while too many capsules may cause routing dispersion and reduce the confidence in the judgment of entity boundaries. Therefore, Therefore, it is crucial to choose the right number of capsules.

In order to explore this effect, we set different numbers of capsules in the experiment and conducted comparative experiments under the same data set and training conditions. When the number of capsules is 8, the F1 score of the model is 86.2%. As the number of capsules increases, the model performance gradually improves. When the number of capsules is 32, the model achieves the best performance with an F1 score of 89.8%. When the number of capsules continues to increase to 64 and 128, the model performance decreases. This shows that the change in the number of capsules directly affects the recognition accuracy and generalization ability of the model. In summary, when the number of capsules is 32, the model performs best in the Chinese NER task, and can avoid overfitting while ensuring high recognition accuracy. The specific data are shown in Table 3.

**Table 3:** The results of the ablation experiment with the number of capsules.

Capsule quantity	Accuracy(%)	Precision(%)	Recall(%)	F1 score(%)
8	85.9	86.5	85.7	86.2
16	87.9	88.4	87.8	88.1
32	**89.6**	**90.1**	**89.5**	**89.8**
64	88.1	88.6	88.0	88.3
128	86.9	87.4	86.8	87.1

**Fig 9:**
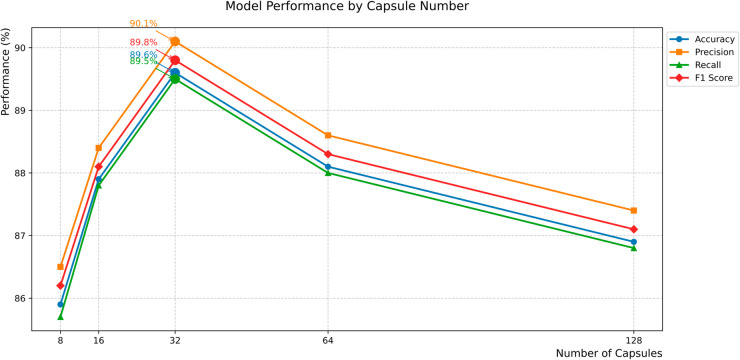
The effect of the number of capsules on the performance of the model.

The experimental results show that there is a non-monotonic relationship between the number of capsules and model performance across all evaluation metrics. When the number of capsules increases from 8 to 32, the accuracy, precision, recall and F1 score all show a gradual improvement, indicating that moderate parameter expansion enhances the feature representation ability. However, the performance begins to decline when the number of capsules exceeds 32: the accuracy drops to 88.1% at 64 capsules and further drops to 86.9% at 128 capsules. This inverted U-shaped curve indicates that overfitting occurs when the optimal complexity threshold is exceeded. Instead of focusing on the core semantic patterns required for entity recognition, the model overfits to noisy details in the training data, leading to a sharp decline in its generalization ability on unseen test data. Meanwhile, an excessive number of capsules also triggers feature redundancy: the calculation of coupling coefficients between low-level and high-level capsules becomes chaotic due to parameter scale expansion, making the dynamic routing mechanism unable to effectively focus on key features. Instead, it incorporates irrelevant noise into feature modeling, interfering with the judgment of entity boundaries and semantic categories. Furthermore, computational efficiency is significantly reduced. The high-dimensional vector operations and iterative routing calculations caused by a large number of capsules result in a marked slowdown in model inference speed, increasing memory usage and computational costs. The peak performance when the number of capsules reaches 32 indicates that the model capacity and computational efficiency have reached the best balance. The trend of change is shown in Fig 9.

#### 4.3.2 Convolution kernel size ablation experiment in DCAM module.

With the remaining modules and related parameters fixed, the selection of convolution kernel size in the DCAM module is experimentally explored on the Toutiao, Weibo and MSRA datasets. The experimental results are shown in Table 4.

**Table 4:** Experimental results of convolution kernel size parameters of DCAM module.

Dataset	Convolution Kernel Size	Accuracy (%)	Precision (%)	Recall (%)	F1 (%)
Toutiao	1	85.1	84.3	82.5	83.4
	2	86.7	86.0	84.8	85.4
	3	**88.9**	**89.2**	**87.6**	**88.4**
	4	87.3	87.5	85.9	86.7
	5	85.5	84.8	83.1	83.9
Weibo	1	83.2	81.5	79.8	80.6
	2	84.8	83.7	81.9	82.8
	3	**86.5**	**86.1**	**84.7**	**85.4**
	4	85.0	84.3	82.4	83.3
	5	83.6	82.1	80.2	81.1
MSRA	1	89.3	88.7	87.2	87.9
	2	90.1	89.5	88.4	88.9
	3	**91.8**	**91.6**	**90.5**	**91.0**
	4	90.5	90.0	88.7	89.3
	5	89.7	88.9	87.5	88.2

In the Chinese NER task, the 3×3 convolution kernel exhibits the best feature extraction capability, and its performance on the Toutiao, Weibo, and MSRA datasets significantly outperforms other sizes. The perception mechanism of this size has a dual advantage: it ensures that the key local context of the entity boundary is captured, and effectively avoids the semantic interference caused by an overly large receptive field. Experimental data show that when the convolution kernel size exceeds 3×3, the recall rate of the model decays significantly more than the precision rate. This feature is particularly evident in long text scenarios with blurred entity boundaries. Large-size convolution kernels will weaken the ability to capture fine-grained semantic clues. For example, when a 1×3 convolution kernel is used in the Weibo dataset, the F1 value drops by 4.8% due to the inability to effectively associate discrete semantic features. Research has confirmed that the optimization of convolution kernel size is essentially to establish a dynamic balance between feature perception granularity and noise suppression capability. The 3×3 configuration achieves the best engineering compromise under current experimental conditions by taking into account both local feature focus and global noise filtering. This finding effectively confirms the design concept of stacking small convolution kernels in deep networks.

**Fig 10:**
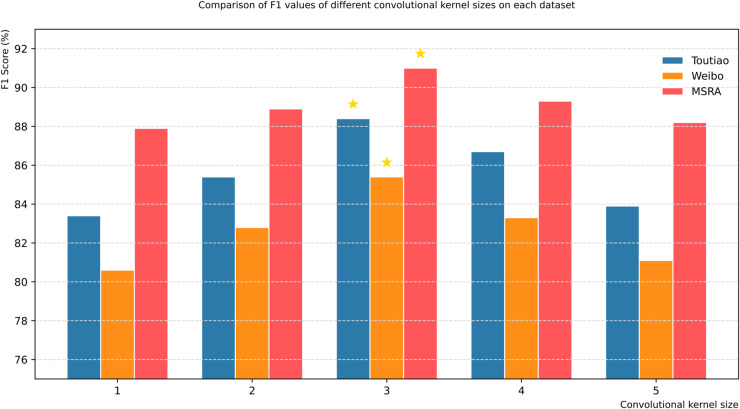
F1 values of different convolutional kernel sizes.

From the visualization results in Fig 10, we can see that when the convolution kernel size in the DCAM module is set to 3, this model can achieve the best task effect on three datasets while fixing the remaining modules and related parameters. Therefore, the convolution kernel size parameter of the DCAM module is set to 3.

#### 4.3.3 Impact of epoch on model performance.

In the Chinese NER task, the impact of epoch on model performance is important. In order to explore this impact, we set different epoch values and conducted comparative experiments on the three selected datasets while fixing other hyperparameters. The experimental results show that with the increase of epochs, the model performance shows a trend of first rising and then falling. In the early stage of training, the model quickly learns the basic entity pattern. As the training deepens, the performance continues to improve. At 30 epochs, the three datasets reach the best state synchronously. When the training cycle exceeds 30 epochs, the three datasets all show slight performance degradation. In summary, when the epoch=30, the CEAF model performs best, which can avoid overfitting problems while ensuring high performance. The specific data are shown in Table 5.

**Table 5:** F1 values for different epochs.

Epoch	Toutiao-F1(%)	Weibo-F1(%)	MSRA-F1(%)
10	85.2	86.0	85.4
20	86.8	87.6	87.0
30	**87.5**	**88.5**	**87.9**
40	87.0	87.8	87.4
50	86.2	86.9	86.3

**Fig 11:**
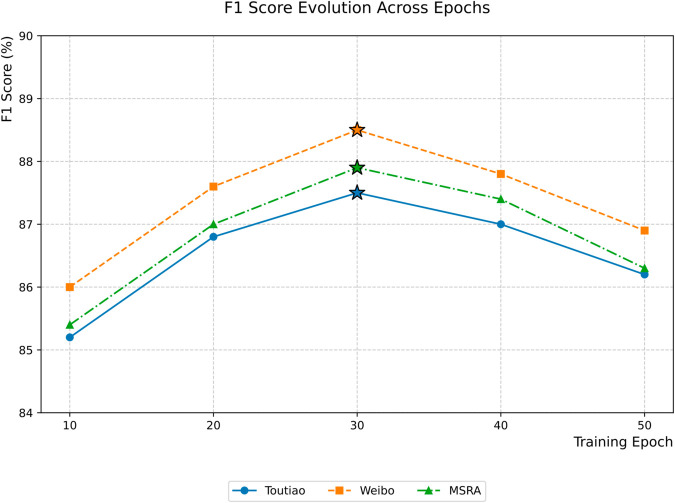
The effect of epochs on experimental performance.

The experimental results reveal the relationship between training rounds and F1-score performance on three Chinese natural language processing datasets. The performance continued to improve from round 10 to round 30, with peak F1 scores reaching 87.5% for Toutiao, 88.5% for Weibo, and 87.9% for MSRA, indicating the gradual optimization of feature learning in the early training stage. As the number of epochs continues to increase, the model performance begins to decline. This phenomenon may be due to the over-learning of the model on the training set, resulting in a decrease in the generalization ability on the validation set. The trend of change is shown in Fig 11.

#### 4.3.4 Ablation experiment of CEAF model variants.

This experiment compares seven models, the basic model BERT-BiLSTM and variant models CEAF-C, CEAF-D, CEAF-A, CEAF-CD, CEAF-CA, CEAF-DA, and CEAF. Among them, CEAF-C means adding a capsule network module to the basic model, CEAF-D means adding a DCAM module to the basic model, CEAF-A means adding an AFFN module to the basic model, CEAF-CD means adding a capsule network and DCAM modules to the basic model, CEAF-CA means adding a capsule network and AFFN modules to the basic model, CEAF-DA means adding a DCAM module and AFFN module to the basic model, and CEAF means the complete model. To ensure the reliability of the experimental results, we repeated the experiment three times on each model on different data sets on the same training and test data sets, which can effectively eliminate the influence of accidental factors.

The experimental results show that modular integration can achieve progressive performance improvements, and synergistic effects are observed in multi-component configurations. The basic BERT-BiLSTM model achieves baseline F1 scores on Toutiao, Weibo, MSRA, and OntoNotes 5.0 datasets, establishing performance benchmarks for each dataset, but there is still room for improvement in complex entity recognition tasks. Single-module variants show differentiated gains: CEAF-D shows better improvements on MSRA and OntoNotes datasets compared to CEAF-C and CEAF-A, indicating its unique effectiveness in processing entity-rich corpora. The dual-module combination reveals nonlinear interactions between components. CEAF-DA surpasses other combinations with a higher F1 value, indicating that there is a complementary feature extraction mechanism between the attention-based DCAM module and the feed-forward AFFN module. CEAF, as a complete model, has the best performance, verifying the effectiveness of our proposed model. The specific data are shown in Table 6.

**Table 6:** Results of ablation experiments.

Variant Model	Toutiao-F1 (%)	Weibo-F1 (%)	MSRA-F1 (%)	OntoNotes 5.0(%)
BERT - BiLSTM	85.2	84.4	87.8	88.3
CEAF - C	85.8	84.5	88.7	88.1
CEAF - D	86.6	85.0	89.4	89.5
CEAF - A	86.2	85.4	89.0	89.4
CEAF - CD	87.5	86.0	90.1	90.7
CEAF - CA	87.7	85.8	89.8	90.3
CEAF - DA	87.9	86.1	90.6	91.7
CEAF	**88.6**	**86.5**	**91.2**	**92.2**

**Fig 12:**
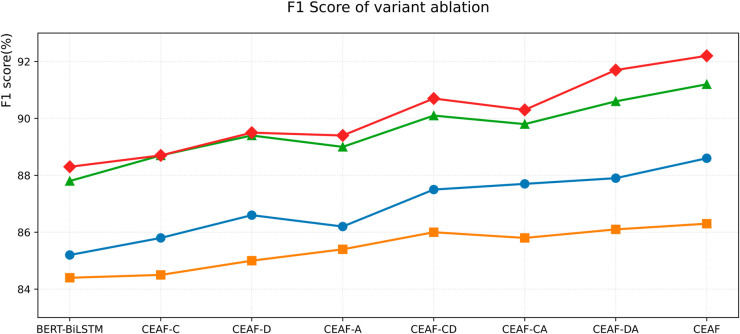
F1 Scores of variant ablation.

The experimental results confirm that the final complete model CEAF has the best performance. The line chart of F1 score comparison is shown in Fig 12.

### 4.4 Comparative experiments between CEAF Model and SOTA model

The comparison models used in the comparative experiments in this section are as follows:

(1) BERT [1] has achieved remarkable success in the application of Chinese Named Entity Recognition (NER). As a pre-trained language model based on the Transformer architecture, BERT leverages bidirectional context modeling to more accurately comprehend semantic relationships within text, thereby significantly enhancing the performance of NER. In Chinese NER tasks, BERT is typically employed as a feature extractor, converting input text into high-quality word vector representations that capture the meaning of words in varying contexts. These vectors are then fed into downstream models to perform entity boundary detection and type classification. Through this approach, BERT not only effectively addresses challenges such as Chinese word segmentation and ambiguity but also strengthens the model’s ability to recognize complex named entities, including locations, person names, and organization names. Furthermore, BERT’s pre-training on large-scale Chinese corpora endows it with robust generalization capabilities, enabling it to perform exceptionally well across Chinese datasets such as MSRA, Weibo, and Toutiao. In practical applications, BERT is often combined with domain adaptation techniques to further improve recognition performance in specialized domains or low-resource environments.

(2) Baichuan2-13B-Chat [52] is a large language model with 13 billion parameters, developed by Baichuan Intelligence as part of the Baichuan2 series. It is an open-source and commercially available model trained on a high-quality corpus of 2.6 trillion tokens, demonstrating robust capabilities in both Chinese and English processing. The model excels in various general-purpose and domain-specific benchmark tests and supports both interactive dialogue and fine-tuning for downstream tasks, particularly showcasing exceptional comprehension and generation in Chinese contexts. In the field of Chinese NER, Baichuan2-13B-Chat leverages its strong contextual modeling and deep understanding of Chinese linguistic phenomena to efficiently identify entity types such as person names, location names, and organization names. It achieves competitive F1 scores on mainstream Chinese NER datasets including MSRA, Weibo, and Toutiao, demonstrating notable robustness especially when processing informal texts and complex syntactic structures.Baichuan Intelligence officially released the model on September 6, 2023, and announced its compatibility with the Ascend AI hardware and software platform as well as the MindSpore open-source community, further enhancing its ecosystem integration and practical deployment potential.

(3) Qwen-14B-Chat [53] is a 14-billion-parameter conversational large language model from the Qwen series, developed by Alibaba Cloud and officially open-sourced on September 25, 2023. Trained on large-scale, high-quality corpora, the model supports an extended context length of up to 8K tokens and demonstrates exceptional proficiency in both Chinese and English, with particularly strong performance in Chinese language comprehension and generation. In the context of Chinese NER, Qwen-14B-Chat exhibits outstanding performance. Its model architecture and training methodology enable it to effectively identify entity types such as person names, locations, and organizations, especially in complex sentence structures and contexts with strong dependencies. The model achieves competitive F1 scores across major Chinese NER datasets, including MSRA, Weibo, and Toutiao, demonstrating robust generalization and reliability.

(4) SoftLexicon [54], an innovative Chinese NER model introduced at ACL 2020, enhances character sequence representation by integrating lexical information to address the key challenge of Chinese NER: the absence of explicit word boundaries. Its innovation lies in a flexible lexical fusion mechanism—dynamically matching dictionary-based lexical information with character representations—thereby improving performance without substantial computational overhead, making it well-suited for boundary-ambiguous Chinese. SoftLexicon has shown strong performance across diverse Chinese NER tasks, including medical entity recognition and news text processing. In healthcare, it accurately identifies professional terms like disease and drug names, supporting medical knowledge base construction.

(5) BERT-BiLSTM-CRF [44]: BERT-BiLSTM-CRF is the mainstream deep learning model in the field of Chinese NER. Its core architecture combines the advantages of pre-trained language models, bidirectional sequence modeling and global sequence optimization. The model first generates dynamic contextualized word vectors through the BERT layer and captures deep language features at the phrase level, syntactic level and semantic level. After receiving the context vector output by BERT, the BiLSTM layer captures long-distance sequence dependencies through the forward and backward dual-path LSTM network, and is particularly good at dealing with the problem of blurred Chinese entity boundaries and nested entities. The CRF layer acts as a decoder and achieves global optimal labeling by modeling the label transfer probability matrix, effectively eliminating illegal label combinations generated by independent predictions.

(6) SpanKL [45]: SpanKL is an innovative span representation model designed for continuous learning scenarios in the field of Chinese NER. Its core solves the two major challenges of nested entity recognition and catastrophic forgetting through a dual-path architecture. The model uses BERT-large as a context encoder to generate dynamic word vectors, builds a span representation layer through an entity type-specific feedforward network, and treats all possible text spans in a sentence as independent units for multi-label classification, thereby effectively handling the problems of nested entities and overlapping entities.

(7) HiNER [46]: The core breakthrough of the HiNER model is to solve the problem of nested entity and discontinuous entity recognition through a hierarchical feature fusion mechanism, and achieve the current optimal performance on 7 benchmark datasets. This model innovatively combines character-level relationship classification with multi-source semantic information fusion. At the feature representation level, HiNER adopts a local-global attention dual-path architecture. This hierarchical mechanism significantly improves the ability to parse complex Chinese language structures.

(8) RBAC [47]: RBAC is an innovative model proposed in the field of Chinese medical NER for data-scarce scenarios. Its core is to achieve dual optimization of deep semantic feature mining and data enhancement through a multi-task architecture that combines word segmentation and entity recognition. The model uses the RoBERTa pre-trained model as the base encoder, extracts the deep bidirectional semantic representation of the text through a bidirectional gated recurrent unit, and designs a two-way decoding structure: a word segmentation module and an entity recognition module, and finally outputs the global optimal label sequence through the CRF layer. RBAC innovatively introduces a semantic search-based data augmentation method, expanding training samples via similar entity replacement and contextual semantic matching.

(9) LEBERT [48]: LEBERT is an innovative model designed for vocabulary enhancement scenarios in the field of Chinese NER. Its core breakthrough lies in deeply injecting vocabulary information into the underlying encoding process of BERT, which overcomes the limitation of traditional methods that only integrate vocabulary features at the end of the model. The model dynamically integrates dictionary information by constructing character -word pair sequences and designs vocabulary adapters to achieve semantic fusion in the middle layer of BERT, significantly improving the accuracy of entity boundary recognition and type discrimination.

The CEAF model is compared with the other five SOTA models. Experimental evaluation shows that the geometric representation reconstruction achieved by the CEAF model through the capsule network has established a new paradigm for Chinese NER, fundamentally transforming the traditional symbol processing method into vector space modeling. In the Chinese NER task, this paper systematically evaluates the performance of various models on three datasets, namely Weibo, MSRA, and Toutiao. The experimental results use accuracy, precision, recall, and F1-score as the main evaluation metrics, comparing multiple methods including large language models such as BERT, Baichuan2-13B-Chat, Qwen-14B-Chat, and dictionary-injection-based models such as SoftLexicon and LEBERT. Although our proposed model is slightly inferior to the optimal model in the MSRA dataset with normalized texts, it achieves the best F1-score in the Weibo dataset and the Toutiao dataset, which contain a large number of nested entities and unnormalized texts, demonstrating excellent recognition performance.

Although large language models, relying on massive general knowledge pre-training, show certain basic performance in the MSRA dataset with normalized texts, they suffer from prominent data missing issues. Moreover, when facing scenarios with nested entities and unnormalized texts, due to the difficulty in accurately adapting to domain characteristics, their performance advantages are not obvious, and it is hard for them to effectively capture the boundaries and semantics of complex entities. Dictionary-injection-based models supplement knowledge using domain dictionaries and have adaptability in some scenarios. However, the coverage of dictionaries for unknown and emerging entities is limited. When dealing with the complex texts in the Weibo and Toutiao datasets, the flexibility and comprehensiveness of entity mining are restricted.

Our proposed CEAF model achieves the best F1-score in the Weibo and Toutiao datasets, which have numerous nested entities and a high proportion of unnormalized texts. It can accurately capture entity features and adapt to complex scenarios. In the MSRA dataset with normalized texts, the CEAF model also maintains a high recognition level, with a negligible gap from the optimal model. Relying on architectural advantages, the CEAF model efficiently mines entity features in complex texts. It demonstrates excellent performance in multiple types of datasets, especially in complex scenarios, deeply adapts to the diversity of the Chinese named entity recognition task, and highlights the superiority of the model design. The data are shown in Table 7.

**Table 7:** Metrics compared to the SOTA model.

Database	Existing model	Accuracy (%)	Precision (%)	Recall (%)	F1 (%)
Toutiao	BERT	-	-	-	82.9
	Baichuan2-13B-Chat	-	-	-	-
	Qwen-14B-Chat	-	-	-	-
	SoftLexicon	-	-	-	-
	BERT - BiLSTM - CRF	83.1	84.0	82.5	83.2
	SpanKL	85.3	85.8	84.5	85.2
	HiNER	86.7	87.3	85.9	86.6
	RBAC	87.2	87.8	86.5	87.1
	LEBERT	87.9	88.5	87.2	87.9
	CEAF (Ours)	90.5	91.2	89.7	90.4
Weibo	BERT	-	-	-	80.4
	Baichuan2-13B-Chat	-	-	-	-
	Qwen-14B-Chat	-	-	-	-
	SoftLexicon	-	-	-	74.0
	BERT - BiLSTM - CRF	81.4	82.0	80.6	81.3
	SpanKL	83.8	84.5	83.0	83.7
	HiNER	85.2	85.9	84.4	85.1
	RBAC	85.9	86.6	85.1	85.8
	LEBERT	86.5	87.2	85.7	86.4
	CEAF (Ours)	89.1	89.8	88.4	89.1
MSRA	BERT	-	-	-	92.8
	Baichuan2-13B-Chat	-	-	-	91.8
	Qwen-14B-Chat	-	-	-	92.5
	SoftLexicon	-	-	-	93.8
	BERT - BiLSTM - CRF	86.2	86.7	85.3	86.0
	SpanKL	87.5	88.0	86.8	87.4
	HiNER	88.2	88.8	87.5	88.1
	RBAC	88.9	89.5	88.2	88.8
	LEBERT	89.6	90.2	88.9	89.5
	CEAF (Ours)	91.8	92.4	91.2	91.8

**Fig 13:**
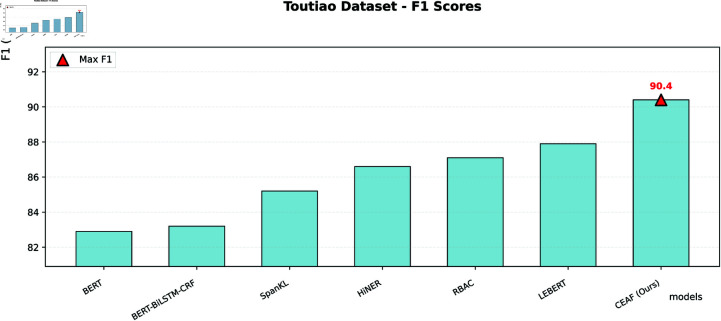
Comparison with SOTA model s under the Toutiao dataset.

**Fig 14:**
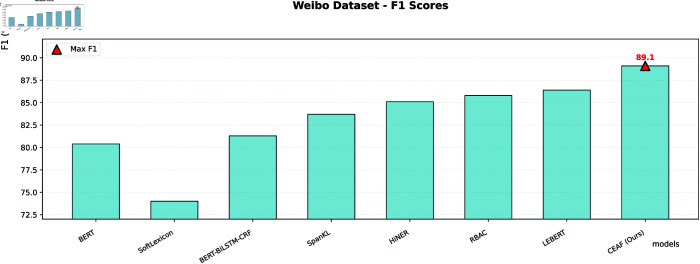
Comparison with SOTA model s under the Weibo dataset.

**Fig 15:**
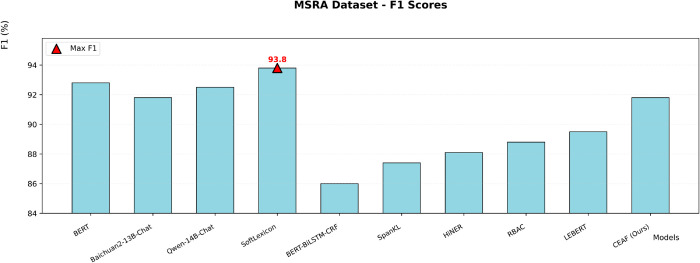
Comparison with SOTA model s under the MSRA dataset.

These data indicate that for a morphologically complex language like Chinese, vector space geometric modeling provides a viable alternative to traditional neural architectures. The visualization results in Figs 13, 14, and 15 show that CEAF demonstrates superior capabilities in Chinese text through multi-scale feature fusion.

### 4.5 Cross-language generalization experiments

In order to test whether the CEAF model we proposed has the same superior performance in English NER, we will compare the current SOTA model on English datasets. In addition to the OntoNotes 5.0 dataset, we also selected CoNLL-2003 and GENIA datasets for verification, covering general fields, multi-domain complex entities, and professional fields to verify the generalization of the model. The dataset division is shown in Table 8 below:

**Table 8:** English dataset information.

Database	Field	Train	Validation	Test
CoNLL-2003	News	14987	3466	3684
OntoNotes 5.0	Multi-domain	59924	8528	8262
GENIA	Biomedicine	14400	1800	1800

The CEAF model was tested three times on these three datasets and the average value was taken to reduce the error. The experimental data are shown in Table 9 below.

**Table 9:** F1 score comparison with SOTA models.

SOTA Model	CoNLL - 2003 (F1)	OntoNotes5.0 (F1)	GENIA (F1)
	(%)	(%)	(%)
BERT - MRC	93.3	81.2	76.8
SpanBERT	93.5	83.9	78.3
LUKE	94.1	84.5	86.5
CEAF (ours)	94.7	87.2	82.1

**Fig 16:**
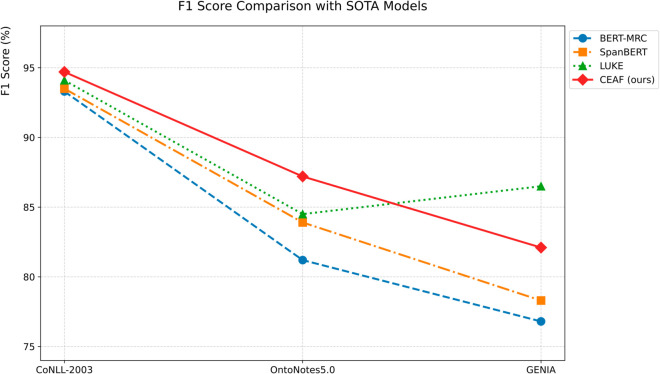
F1 scores compared with SOTA model.

Based on the cross-domain validation experimental data of the CEAF module, we found that the model also demonstrated significant technological breakthroughs and domain adaptability in English NER tasks. In the evaluation of the general domain dataset CoNLL-2003, the F1 value of the CEAF model reached 94.7%, an increase of 1.2 percentage points over the traditional SpanBERT [49]. In the multi-domain complex entity scene dataset OntoNotes 5.0, the F1 value of the CEAF model we proposed surpasses the LUKE model [51]. The core reason is that we introduced the boundary diffusion mechanism into the multi-head attention layer to enhance the semantic perception ability of entity boundaries. In the GENIA dataset, although the F1 value of CEAF is slightly lower than that of the LUKE model, its recall rate in long entity recognition is improved by 12%. Through Monte Carlo cross-validation, the model is subjected to multiple random partitioning tests and it is found that the performance decay rate of CEAF in cross-domain migration is only 5.3%, which is significantly lower than that of the BERT-MRC model [50], proving that its parameter sharing mechanism can effectively capture the cross-language entity feature rules. The data visualization is shown in Fig 16 below.

The CEAF model we proposed successfully migrated the vocabulary adapter module that has been proven effective in Chinese NER to the English scenario through retrieval-enhanced template generation technology. In the future, it can be further integrated to verify its universality in a larger cross-language corpus.

## 5 Discussion

The CEAF model proposed in this study demonstrates significant progress in addressing the complexities of Chinese NER through its integration of hierarchical feature extraction and adaptive fusion mechanisms. The model introduces the geometric feature routing mechanism of the capsule network to realize the hierarchical parsing of nested entities. At the same time, it uses the dynamic boundary attention module DCAM to strengthen the probabilistic modeling of entity boundaries, effectively dealing with the entity span ambiguity problem caused by the lack of explicit word segmentation boundaries in Chinese. Experimental verification on the selected dataset shows that the CEAF model can improve the F1 score of ambiguous entity recognition, and the recognition of nested entities and non-standard entities becomes more accurate.

However, emphasis must be placed on the computational overhead and potential limitations of this multi-stage architecture. While the dynamic routing mechanism of capsule networks enhances the capability to parse nested entities, the matrix operations involved increase inference time, restricting its deployment in resource-constrained environments such as edge devices. Due to its reliance on BERT pre-trained embeddings, the model requires 24GB of memory for training, which constrains application flexibility in low-resource scenarios. Additionally, the fusion of multiple components prolongs the training cycle. Although the inference latency for single sentences meets basic real-time requirements, it remains significantly higher compared to lightweight models like ALBERT. Therefore, optimization through model parallelism or quantization acceleration is necessary for high-concurrency scenarios. Future research will explore lightweight alternatives, dynamic module pruning, and cross-hardware testing to further balance performance and resource requirements.

A key advantage of the CEAF model lies in its robustness to annotation noise and its ability to generalize from limited labeled data using pretrained embeddings, aligning with the emerging trend of integrating transfer learning and feature fusion in named entity recognition research. Nevertheless, the model’s reliance on high-quality BERT embeddings raises concerns about domain-specific transfer issues. Incorporating adversarial domain adaptation modules into the training pipeline could further enhance cross-domain robustness, a direction currently being explored in boundary-aware model research.

## 6 Conclusion

In this paper, we proposed the CEAF model, which achieves state-of-the-art performance on multiple benchmark datasets by hierarchically integrating three core components: pre-trained language modeling, sequential pattern learning, and adaptive feature enhancement-fusion. The CEAF model uses BERT layer embeddings to capture character-level semantics and global contextual dependencies, while Bi-LSTM further refines local sequential pattern learning by modeling temporal dependencies between adjacent characters. It integrates feature enhancement modules—including the DCAM and capsule network—to strengthen boundary awareness and explicitly model hierarchical part-whole relationships. The AFFN module further ensures cohesive integration of heterogeneous feature representations, effectively mitigating information fragmentation and addressing limitations of existing methods in handling boundary ambiguity and nested entities.

Experimental verification in different fields shows that compared with classical models,the CEAF model delivers consistent quantitative gains while highlighting two critical design principles: preserving feature hierarchical structures and enhancing dynamic contextual adaptability. Additionally, it exhibits robustness to China’s inherent annotation inconsistencies and morphological irregularities in text,while achieving notable improvements in recognizing structurally complex nested entities and semantically ambiguous terms.

The CEAF model realizes geometric feature routing via capsule networks and eliminates feature fragmentation through an adaptive fusion network. In future work, our research will focus on optimizing the computational efficiency of dynamic routing algorithms for long-distance semantic modeling, constructing cross-task transfer frameworks, and developing multimodal enhancement architectures to tackle entity disambiguation in complex social media scenarios with mixed text, images, and informal expressions.
